# Supplementary material for: Effectiveness of exercise intervention during pregnancy on high-risk women for gestational diabetes mellitus prevention: A meta-analysis of published RCTs
Source: PLoS One. 2022 Aug 5;17(8):e0272711. doi: 10.1371/journal.pone.0272711 (PMC9355219; doi:10.1371/journal.pone.0272711)
Supplement: S1 Table — (DOCX) [file pone.0272711.s001.docx]

**S1 Table. Search strategy**

| PubMed | (exercise OR “physical activit*” OR workout) AND (diabetes AND (gestational OR pregnancy)) AND ("Clinical Trials as Topic"[Mesh] OR "randomized controlled trial"[pt] OR "controlled clinical trial"[pt] OR randomized[tiab] OR placebo[tiab] OR randomly[tiab] OR trial[tiab]) |
| --- | --- |
| CENTRAL | (exercise OR “physical NEXT activit*” OR workout) AND (diabetes AND (gestational OR pregnancy)) (Word variations have been searched) |
| Scopus | TITLE-ABS-KEY ( ( exercise OR "physical activit*" OR workout ) AND (diabetes AND ( gestational OR pregnancy ) ) ) AND ( LIMIT-TO ( DOCTYPE , "ar" ) ) AND ( LIMIT-TO ( EXACTKEYWORD , "Female" ) OR LIMIT-TO ( EXACTKEYWORD , "Pregnancy" ) ) AND ( LIMIT-TO ( LANGUAGE , "English" ) ) |
