# Supplementary material for: Effectiveness of exercise intervention during pregnancy on high-risk women for gestational diabetes mellitus prevention: A meta-analysis of published RCTs
Source: PLoS One. 2022 Aug 5;17(8):e0272711. doi: 10.1371/journal.pone.0272711 (PMC9355219; doi:10.1371/journal.pone.0272711)
Supplement: S2 Table — (DOCX) [file pone.0272711.s002.docx]

**S2 Table. Evaluation of the exercise intervention based on CERT tool**

| **First author, Publication year, Country** | **1)Equipment** | **2)Provider** | **3)Individually or in a group** | **4)Supervised or unsupervised** | **5)Adherence to exercise** | **6)Motivation strategies** | **7)Decision rules for progressing the exercise program** | **8)Description of each exercise, reproduction** | **9)Content of any home program component** | **10)Nonexercise components** | **11)Adverse events documentation and management** | **12)Location** | **13)Dosage** | **14)Generic or tailored to the individual** | **15)Decision rule for determination of the starting level for exercise** | **16)Whether the exercise intervention delivered and performed as planned** |
| --- | --- | --- | --- | --- | --- | --- | --- | --- | --- | --- | --- | --- | --- | --- | --- | --- |
| Nicolette Oostdam 2012 Netherlands | Cycle ergometers, treadmills, cross-trainers, stationary rowing machines, free weights, accelerometer. | Physiotherapist | Individually | Supervised | Accelerometer, MET from the American College of Sports Medicine statement (ACSM). | Information on the benefits for mother and child at the start and during the intervention. | The training load will be increased when the desired number of repetitions with the current load is reached. A check to avoid overexertion will be the "talk test". The physiotherapist increases the work load till an intensity is reached that equals a Borg scale rating of 12. When the RPE is rated under 12, the intensity will be increased by increasing the working load. | ND | ND | ND | No adverse events resulting from the intervention were reported. | Department of Physiotherapy in the participating hospitals  (VUmc, OLVG, SLAZ, MST, and Isala). | Each exercise session will begin with a warming-up for 5–10 minutes. This is a light intensity activity, such as slow cycling at a level of intensity of 50 Watt. After the warming-up, each participant completes an individualised program of 40 minutes, consisting of 1 or 2 aerobic exercises and 4 to 6 strength exercises. After exercising, participants cool down for 5–10 minutes by slowly reducing the activity. | The training intensity was carefully and individually controlled. | At the start of the intervention exercises will be adjusted to maximal muscle strength and aerobic capacity of the women. The one Repetition Maximum (1-RM; the heaviest weight that can be lifted only once) weight lifting test to measure muscular strength cannot be used during pregnancy. Woman should avoid overly vigorous activity, and the Valsalva maneuver during resistance exercise. Therefore 1RM for each strength exercise will be predicted from the Oddvar Holten diagram. | Training will take place under close supervision of a physiotherapist. The physiotherapists will use the guide of the American College of Obstetricians and Gynaecologists to maintain a safe and healthy exercise program. |
| Bradley B. Price  USA | Treadmills, elliptical trainers, stationary bicycles, weight  machines, exercise balls | Author | Both as a group and individually | Supervised | All study participants underwent five fitness assessments. Each participant estimated perceived exertion on the Borg Scale after walk or run 3.2 km (2 miles) as fast as possible within her comfort zone at a steady pace.  Temperature and humidity rates were documented during each fitness assessment. The sit-and-reach test was used to assess hamstring and trunk flexibility. Before every fitness assessment, subjects completed a 36-item Maternal Physical Discomfort Scale, a psychometrically validated questionnaire designed to measure discomfort in pregnancy. | ND | A program at moderate intensity (12–14 on Borg Scale of perceived exertion). | ND | ND | All subjects were told to follow the dietary advice of their obstetricians or  midwives, and there was no attempt to estimate calorie intake. | Intervention Group -anxiety with exercise (n = 1) -history of preterm pregnancy (n = 1) -pain from leiomyomas (n = 1) | ND | Aerobic training of 45–60 min duration, performed 4 times per  week, at moderate intensity (12–14 on Borg Scale of perceived exertion), consistent with exercise guidelines of the ACOG. Step aerobics on the first day, walked as a group on the second day, and circuit training on a third day, consisted of 1–10 min of aerobic exercise, alternating with an equal time interval of weight training, using a weight that allowed one set of 20 repetitions.  The circuit session ended with 5 min of hamstring, quadriceps, and calf stretching. Each woman also  completed a brisk 30- to 60-min walk individually once weekly. | Generic | Before the aerobic fitness test, all study participants per formed a submaximal strength assessment by lifting a 7-kg  medicine ball from floor to waist high as many times possible within 1 min. | The author kept a log of exercise activities and attendance. |
| Ruben Barakat  2013  Spain | Bar-bells, therabands, heart rate (HR) monitor | Qualified fitness specialist with the assistance of an  obstetrician. | In a group | Supervised | Women used a heart rate (HR) monitor during the training sessions to ensure that exercise intensity was moderate; Intensity was also controlled by Borg’s conventional (6–20 point) scale. | All sessions were accompanied with music, and were performed in an airy, well-lighted exercise room at the Hospital. | ND | ND | ND | ND | Under medical follow-up  throughout the entire pregnancy period in the same obstetrics hospital department (Hospital  Severo Ochoa, Madrid, Spain). Usual visits with healthcare providers (midwives, obstetricians and family doctors) during pregnancy. | In an airy, well-lighted exercise room at the Hospital. | 3 days/week, 50–55 min/ session from weeks 10 to 12 of pregnancy to the end of the third trimester. We also included in the programme one session/week of aerobic dance, using choreography involving the upper and lower body limbs of very low impact. Aerobic dance activities  were developed in sections of 3–4 min with 1 min breaks which included stretching and relaxation activities. The main part of the session was preceded and  followed by a gradual warm-up and cool-down period, respectively, both of 10–12 min duration and consisting of walking and light, static stretching of most muscle  groups. The main part of the session lasted 25–30 min and included moderate-intensity resistance exercises. Resistance exercises were performed through the full range of motion normally associated with correct technique for  each exercise and engaged the major muscle groups. It included one set of pelvic tilting in standing position and exercises using bar bells or low-to-medium resistance (elastic) bands (Therabands). | One size fits all. | ND | A qualified fitness specialist carefully supervised every training session with the assistance of an obstetrician and worked with groups of 10–12 women. |
| Carrie Nobles  2015  USA | Digital pedometer | Health educators | ND | ND | Questionnaires | Booster telephone calls and tip sheets mailed. | Weekly goals increasing time spent in moderate intensity physical activity by 10% to safely progress toward the overall activity goal. | ND | ND | Counseling of Institute of Medicine guidelines for appropriate nutrition and weight gain in pregnancy. | Information on adverse birth outcomes abstracted from medical records. | ND | 30 minutes or more of moderate-intensity physical activity on most days of the week. Specific activities self-selected and including dancing, walking, and yard work. | ND | At the baseline visit, health educators administering a 65-item tailoring questionnaire, assessing current stage of motivational readiness for physical activity adoption, self-efficacy, decisional balance, use of cognitive and behavioral processes of change, and time spent in physical activity. | Questionnaires-based individually tailored reports mailed to the participant along with the corresponding stage-matched manual. |
| SN Seneviratne New Zealand 2015 | Magnetic stationary bicycles, heart rate monitors. | Exercise physiologist | Individual | Unsupervised | Participants were provided with heart rate monitors to wear during all cycling sessions, and given target heart rates to maintain exercise sessions at moderate intensity. | ND | ND | Participants received a written programme prescribing frequency and  duration of weekly exercises. A total of 67 sessions were prescribed. | A structured homebased moderate-intensity antenatal exercise programme. | ND | There were no adverse effects reported in association with the exercise intervention. | A home-based antenatal stationary cycling  exercise intervention. | Each exercise session included a 5-minute warm-up and cool-down period at low intensity. A total of 67 sessions were prescribed (frequency varying between three and five sessions per week, and duration of moderate-intensity exercise between 15 and 30 minutes per session, according to stage of pregnancy. | According to stage of pregnancy. | ND | An exercise physiologist was available for help with exercise-related problems. The number of sessions completed and duration and intensity of cycling undertaken were obtained by downloading heart rate monitor data. |
| Kym J. Guelfi  2016  Australia | Upright cycle ergometer, accelerometer | Exercise physiologist | Individually | Supervised | Accelerometer, Exercise Habit Strength Questionnaire | ND | The duration of each session was progressively increased by 5-minute increments every 2–3 weeks, as tolerated,  from 20 to 30 minutes to a maximum session duration of 60 minutes.  The degree of progression was dependent on the baseline fitness level of the woman and her ongoing pregnancy symptoms. | ND | A supervised home-based stationary cycling program. | ND | Two women experienced pregnancy loss at 19 weeks of gestation and one at 21 weeks of gestation (control = 2; exercise = 1). | At participants home, Perth, Australia. | Each session commenced with a 5-minute warm up consisting of pedaling at an intensity that equated to 55–65% of age-predicted maximum heart rate and a rating of perceived exertion of 9–11 on the Borg scale. The subsequent conditioning period was divided into 5-min periods of continuous moderate-intensity cycling alternating with 5-min periods of interval cycling. Two types of intervals were used; an increase in pedaling rate for 15 seconds and an increase in cycling resistance for 30 seconds repeated every 2 minutes. A 5-minute cool down concluded each session followed by light stretching. | Tailored to the individual | ND | All sessions were supervised by an exercise physiologist who would travel to the woman’s home three times each week to monitor the duration and intensity of exercise. |
| Kirsti Krohn Garnæs  2016  Norway | Τreadmill | Physical therapist | In a group | Supervised | Reports of participants in a training diary | Motivational interview session, either individually or in a group and encouragement to compare their own weight gain with the recommended | ND | ND | 50 min at least one weekly and daily pelvic floor muscle exercises. | ND | No adverse events reported during the exercise training or study assessments | St. Olvas Hospital | Three times a week 35 minutes of moderate-intensity endurance exercise and 25 minutes of strength training. Determination of the endurance exercise at 80% of the maximum capacity, according to the Borg scale 12-15. | Adjustment of the program to each woman’s strength level. | ND | ND |
| Chen Wang  2017  China | Stationary bike | Research staff | ND | Supervised | Questionnaires and supervisor records based on the RPE score of subjective assessment of exercise intensity and the Borg scale assessing the RPE score. | ND | Progressive increase of the exercise duration to 45-60 minutes, adding 5 minutes to the intervals or the continuous phases of cycling, based on individual abilities | ND | ND | ND | Standard prenatal care for all women. Recording and review of cervical length at each examination and exclusion of women with a cervical length <25 mm at any time during the intervention because uterine cervix length is an accurate predictor of the risk of preterm birth. | Peking University First Hospital | Exercise at the beginning of the intervention at the lower calculated limit, based on the maximum predicted heart rate for age, progressively increased with the progress of the program, at least 3 days a week. | ND | At study entry, measurements of each participant’s height to the nearest 0.5 cm without shoes and weight accurate to 0.1 kg with light clothing. BMI calculation as maternal weight divided by height (kg/m2). | International Physical Activity Questionnaire. Questions about the number of days per week and the time spent sitting, walking, and doing moderate and vigorous activities, and then a score expressed as metabolic equivalents of task min/wk |
| Niamh Daly 2017 Ireland | Weights | Research staff | In a group | Supervised | Measures were taken to improve compliance with the intervention, including goal-setting and journaling of class. Women in the intervention arm also received an invitation to a secret Facebook group to create a sense of community among participants, to share healthy lifestyle advice, and to improve compliance with the exercise intervention. | Classes were designed such that no woman was alone or isolated during a class. A “kids’ corner” with toys and a mat and playpen for babies helped ensure that childcare was not a barrier. Eleven classes were taught each week, on a choice of days, at 7:30 AM, 11:45 AM, and 5:30 PM. Classes varied each day to maintain interest. | Cardiovascular training aimed to  increase maternal heart rates and exertion was monitored with the Borg Scale of Perceived Exertion, as recommended by the American College of Obstetricians and Gynecologist | ND | ND | Secret Facebook group to create a sense of community among  participants, to share healthy lifestyle advice, and to improve compliance with the exercise intervention. As part of routine prenatal care in Ireland, all women receive a pamphlet with information on healthy eating based on our national guidelines for nutrition and pregnancy. | No adverse events were reported for the intervention group. | Coombe Women and Infants University Hospital, Dublin, Ireland | The program consisted of 50–60 minutes of exercise with a 10-minute warm-up, 15–20 minutes of resistance  or weights, 15–20 minutes of aerobic exercises, and a 10-minute cool-down. The warm-up focused on core  and pelvic floor exercises. The resistance session worked muscle groups important for a healthy pregnancy and birth recovery, the lower limb, upper limb, back, and core muscle groups. | Exercises were scaled  according to ability. The potential benefits of participation were outlined, and individual “SMART” (specific, measurable, achievable,  relevant, and time-specific) personal goals were set. | The ParMed-X for pregnancy form was used to rule out contraindications to exercise in pregnancy. | Attendance at the intervention class was recorded by both participants and the researcher. |

ND, no data
