# Supplementary material for: Effectiveness of exercise intervention during pregnancy on high-risk women for gestational diabetes mellitus prevention: A meta-analysis of published RCTs
Source: PLoS One. 2022 Aug 5;17(8):e0272711. doi: 10.1371/journal.pone.0272711 (PMC9355219; doi:10.1371/journal.pone.0272711)
Supplement: S3 Table — (DOCX) [file pone.0272711.s003.docx]

**Table S3**. Subgroup and sensitivity analyses

| **Factor** | **Subgroup** | **Number of studies** | **Odds Ratio**  **(95% CI)** | **P-value** | **Cochran’s Q statistic** | **I^2^**  **(95% CI)** | **Test of difference P-value** |
| --- | --- | --- | --- | --- | --- | --- | --- |
| **Subgroup analyses** | | | | | | | |
| Increased BMI as a risk factor | Included | 6 | 0.68 (0.43, 1.09) | 0.11 | 8.01 | 38% (0, 75%) | 0.78 |
|  | Not included | 3 | 0.74 (0.52, 1.06) | 0.1 | 1.59 | 0% (0, 90%) |  |
| Low education level | More than 5% of participating women | 4 | 0.55 (0.4, 0.74) | 0.0001 | 1.53 | 0% (0, 85%) | 0.97 |
|  | Up to 5% of participating women | 1 | 0.53 (0.16, 1.81) | 0.31 | n/a | n/a |  |
| Motivation component in the intervention | Included | 5 | 0.69 (0.50, 0.96) | 0.03 | 3.46 | 0% (0, 79%) | 0.85 |
|  | Not included | 4 | 0.74 (0.38, 1.47) | 0.39 | 6.57 | 54% (0, 85%) |  |
| Provision of intervention | Individually | 3 | 0.96 (0.58, 1.60) | 0.89 | 1.49 | 0% (0, 90%) | 0.58 |
|  | In group | 3 | 0.78 (0.44, 1.37) | 0.38 | 3.24 | 38% (0, 81%) |  |
| Intervention duration | More than 20 weeks | 5 | 0.54 (0.4, 0.74) | 0.0001 | 1.57 | 0% (0, 79%) | 0.02 |
|  | Up to 20 weeks | 3 | 1 (0.66, 1.5) | 0.99 | 3.04 | 1% (0, 79%) |  |
| **Sensitivity analyses** | | | | | | | |
| Detection bias | Low | 4 | 0.73 (0.49, 1.08) | 0.11 | 3.29 | 9% (0, 86%) | n/a |
| Attrition bias | Low | 8 | 0.7 (0.51, 0.97) | 0.03 | 10.07 | 31% (0, 69%) | n/a |

GDM, gestational diabetes mellitus; OR, odds ratio; CI, confidence interval; n/a, not applicable
