# Supplementary material for: Effectiveness of exercise intervention during pregnancy on high-risk women for gestational diabetes mellitus prevention: A meta-analysis of published RCTs
Source: PLoS One. 2022 Aug 5;17(8):e0272711. doi: 10.1371/journal.pone.0272711 (PMC9355219; doi:10.1371/journal.pone.0272711)
Supplement: S4 Table — (DOCX) [file pone.0272711.s004.docx]

**Table S4**. Meta-regression results for GDM OR

| **Covariate** | **Number of studies** | **Coefficient b (95% CI)** | **P-value** | **tau^2^** |
| --- | --- | --- | --- | --- |
| Baseline risk of GDM | 9 | -0.32 (-3.48, 2.83) | 0.813 | 0.08 |
| Study duration (months) | 9 | -0.005 (-0.037, 0.028) | 0.73 | 0.07 |

GDM, gestational diabetes mellitus; OR, odds ratio; CI, confidence interval
