# Supplementary material for: Effectiveness of exercise intervention during pregnancy on high-risk women for gestational diabetes mellitus prevention: A meta-analysis of published RCTs
Source: PLoS One. 2022 Aug 5;17(8):e0272711. doi: 10.1371/journal.pone.0272711 (PMC9355219; doi:10.1371/journal.pone.0272711)
Supplement: S5 Table — (DOCX) [file pone.0272711.s005.docx]

**Table S5.** GRADE evaluation of overall evidence

| **Exercise for GDM prevention** | | | | | | |
| --- | --- | --- | --- | --- | --- | --- |
| **Patient or population:** pregnant women with high risk for GDM **Settings:** outpatient **Intervention:** Exercise | | | | | | |
| **Outcomes** | **Illustrative comparative risks* (95% CI)** | | **Relative effect (95% CI)** | **No of Participants (studies)** | **Quality of the evidence (GRADE)** | **Comments** |
|  | Assumed risk | Corresponding risk |  |  |  |  |
|  | **Control** | **Exercise** |  |  |  |  |
| **GDM** | **Study population** | | **OR 0.7**  (0.52 to 0.93) | 1508 (9 studies) | ⊕⊕⊕ moderate |  |
|  | **281 per 1000** | **215 per 1000** (169 to 267) |  |  |  |  |
|  | **Moderate** | |  |  |  |  |
|  | **222 per 1000** | **166 per 1000** (129 to 210) |  |  |  |  |
| *The basis for the **assumed risk** (e.g. the median control group risk across studies) is provided in footnotes. The **corresponding risk** (and its 95% confidence interval) is based on the assumed risk in the comparison group and the **relative effect** of the intervention (and its 95% CI).  **CI:** Confidence interval; **OR:** Odds ratio; | | | | | | |
| GRADE Working Group grades of evidence **High quality:** Further research is very unlikely to change our confidence in the estimate of effect.  **Moderate quality:** Further research is likely to have an important impact on our confidence in the estimate of effect and may change the estimate. **Low quality:** Further research is very likely to have an important impact on our confidence in the estimate of effect and is likely to change the estimate. **Very low quality:** We are very uncertain about the estimate. | | | | | | |
|  | | | | | | |
