# Supplementary material for: Effectiveness of exercise intervention during pregnancy on high-risk women for gestational diabetes mellitus prevention: A meta-analysis of published RCTs
Source: PLoS One. 2022 Aug 5;17(8):e0272711. doi: 10.1371/journal.pone.0272711 (PMC9355219; doi:10.1371/journal.pone.0272711)
Supplement: Attachment — Submitted filename: Responses to Editor and Reviewers2.docx [file pone.0272711.s008.docx]

**Responses to Editor and Reviewers**

Editor’s comments

1) The author has added the following to the abstract – however the sentence does not really make sense - However, we could not exclude large variability in study effects (I2 0; 95%CI 0, 90%; and I2 0; 95%CI 0, 85% respectively).

REPLY: We thank the editor for this comment. We rephrased to “However, we could not exclude large variability in study effects because the upper limit of I² confidence interval was higher than 50% for all the analyses.”

2) The author stated that the study was preregistered even though the registration was performed after the first submission.

REPLY: We rephrased to “registered”. (Page 6; Paragraph 1)

3) The quality of the forest plot, funnel plot and the flowchart is still too low for printing.

REPLY: As suggested, we improved the quality of the forest plot, funnel plot and the flowchart.

4) The figures are not sufficiently self-explanatory – most of the figures need more explanation.

REPLY: We thank the Editor for this comment. We have now rephrased the Legends for the three figures to sufficiently explain their content.

5) Figure 1 presenting the search is still insufficient (does not define Mesh and text words for searching in Medline and Central – even though the author stated that the search was updated – but the one added in table one is limited up-to July 2020 and in Scopus studies from 2021 and 2022 is deleted.

REPLY: As suggested, we updated our search up to May 2022. Table S1 includes the exact MeSH terms and keywords, we used. We have also updated Figure 1. The number of articles in the Flowchart (Figure 1) correspond to the numbers we found in our last search (May 2022).

6) Table 3 are showing a subgroup analysis – however presenting the Q, I-square and p-value for the subgroup – however the test of difference may be the one of clinical interest.

REPLY: As suggested, we added the P-value for the test of difference for all subgroup analyses in the revised Table S3.

7) Table 4 is not clear and need more explanation.

REPLY: As suggested, we clarified Table 4, and provided additional explanation “GDM was diagnosed by measuring fasting blood glucose, hemoglobin A1c, or by an oral glucose tolerance test (Table 4).” (Page 14; paragraph 2)

8) Table 5, the SOF table need explanation for what is study population and what does moderate means – the evidence is judged moderate even though the confidence interval is very broad and overlapping 1 (indicating no significant effect).

REPLY: As suggested we clarified “Five out of the 9 studies had potential performance, detection, or attrition bias. The other 4 studies were unclear about blinding. Overall, moderate quality of evidence showed that exercise during pregnancy for the population of women with high risk for GDM may have benefit when compared to standard prenatal care in reducing the risk of GDM. (Table S5)” (Page 17, Paragraph 1)

Reviewer 1

All comments have been addressed.

REPLY: We thank the Reviewer for this comment.

Reviewer 2

1) The protocol is made after the first submission, which is a big limitation. The search is very deficient, and lacks Mesh terms and many relevant keywords, resulting in missing relevant articles (Unless table 1, isn't updated since last submission)

REPLY: We thank the Reviewer for this issue. As suggested, we have updated our initial search strategy including additional MeSH terms and keywords and expanding our search (from inception) to May 2022. We have updated Table S1 to include the exact search strategy. We have also updated all Tables, and Figures in the Results so that they include the additional studies. The text has also been updated as appropriate.

2) The following 3 articles would be relevant to include, and meet your inclusion criteria:

1. Barakat R, Pelaez M, Lopez C, Lucia A, Ruiz JR. Exercise during pregnancy and gestational diabetes-related adverse effects: a randomized controlled trial. Br J Sports Med. 2013 Jul; 47 (10): 630-6. doi: 10.1136 / bjsports-2012-091788. Epub 2013 Jan 30. PMID: 23365418.

(Inclusion criteria included: being sedentary, with match yours)

2. Seneviratne SN, Jiang Y, Derraik J, McCowan L, Parry GK, Biggs JB, Craigie S, Gusso S, Peres G, Rodrigues RO, Ekeroma A, Cutfield WS, Hofman PL. Effects of antenatal exercise in overweight and obese pregnant women on maternal and perinatal outcomes: a randomized controlled trial. BJOG. 2016 Mar; 123 (4): 588-97. doi: 10.1111 / 1471-0528.13738. Epub 2015 Nov 6. PMID: 26542419.

(Participants were women aged 18–40 years with a body mass index (BMI) ≥25 kg / m2,)

3. From Oliveria Melo AS, Silva JL, Tavares JS, Barros VO, Leite DF, Amorim MM. Effect of a physical exercise program during pregnancy on uteroplacental and fetal blood flow and fetal growth: a randomized controlled trial. Obstet Gynecol. 2012 Aug; 120 (2 Pt 1): 302-10. doi: 10.1097 / AOG.0b013e31825de592. PMID: 22825089.

(The inclusion criteria consisted of: healthy pregnant women who were sedentary at admission to the
study)

REPLY: Indeed, we have identified the three papers the Reviewer has indicated. As suggested, we have now added Bakarat et al. 2013, and Seneviratne et al. 2016 in our updated search and analyses. However, we had to exclude Oliveiria Melo et al. 2012 because it did not fulfill our inclusion criteria. Specifically, it did not report the diagnosis of GDM as one of its outcomes.
